# Behavioral and Sociodemographic Predictors of Diabetes Among Non-Hispanic Multiracial Adults in the United States: Using the 2023 Behavioral Risk Factor Surveillance System

**DOI:** 10.3390/ijerph22121815

**Published:** 2025-12-04

**Authors:** Ermias Turuse, Sherli Koshy-Chenthittayil, Amy E. L. Stone, Edom Gelaw, Courtney Coughenour

**Affiliations:** 1 Department of Environmental and Global Health, School of Public Health, University of Nevada, Las Vegas, NV 89154, USA; courtney.coughenour@unlv.edu; 2 Office of Institutional Effectiveness, Touro University Nevada, Henderson, NV 89014, USA; skoshy-c@touro.edu; 3 Department of Medical Education, Kirk Kerkorian School of Medicine at the University of Nevada, Las Vegas, NV 89154, USA; amy.stone@unlv.edu; 4 Department of Epidemiology and Biostatistics, School of Public Health, University of Nevada, Las Vegas, NV 89154, USA; gelawe1@unlv.nevada.edu

**Keywords:** diabetes, behavioral predictors, non-Hispanic multiracial adults

## Abstract

Background: Diabetes disproportionately affects U.S. subgroups, yet non-Hispanic multiracial adults are underrepresented in epidemiologic studies. This study aimed to examine behavioral and sociodemographic predictors of diabetes in this population. Methods: We analyzed data from the 2023 Behavioral Risk Factor Surveillance System (BRFSS) using a cross-sectional design that incorporated survey weights, strata, and primary sampling units. Binary logistic regression was employed to identify predictors of diabetes, including variables with *p* ≤ 0.20 from bivariate models in the multivariable analysis. Adjusted odds ratios (AORs) with 95% confidence intervals (CIs) were reported. Results: The study included a total of 6429 participants. Obesity (AOR = 4.16; 95% CI: 3.33, 33.23), being overweight (AOR = 2.05; 1.62, 2.60), poor general health (AOR = 2.82; 2.38, 38.35), age ≥ 65 years (AOR = 3.08; 2.60, 3.65), male sex (AOR = 1.34; 1.15, 1.58), and health insurance (AOR = 2.14; 1.35, 3.61) were associated with higher odds of diabetes. Physical activity (AOR = 0.76; 0.64, 0.90) and alcohol consumption (AOR = 0.55; 0.47, 47.65) were linked to lower odds of diabetes. Smoking status showed no significant association after adjustment. Conclusions: In non-Hispanic multiracial adults, factors such as adiposity and older age increased the risk of diabetes, while physical activity and alcohol consumption offered protective benefits. These findings indicate that current diabetes prevention strategies are applicable to this subgroup, and public health initiatives should prioritize their inclusion in outreach, screening, and intervention efforts.

## 1. Introduction

Diabetes mellitus is a chronic metabolic disorder marked by persistent high blood sugar levels due to impaired insulin secretion, insulin action, or both [1,2]. It remains a leading cause of morbidity and mortality worldwide [3]. The disease gradually harms essential organs, including the eyes, kidneys, nerves, heart, and blood vessels. This leads to a significant decrease in quality of life and increased healthcare costs [4,5].

In 2021, around 38.4 million people in the United States, or 8.5% of the population, had diabetes [6]. This figure included 38.1 million adults [7], of whom 29.7 million were diagnosed, while approximately 8.7 million remained undiagnosed. Racial and ethnic disparities in diabetes burdens have been well-documented [8], with minority groups experiencing a greater disease burden compared to non-Hispanic Whites [9,10]. These disparities are often linked to a complex interplay of socioeconomic status (SES), behavioral factors, and access to healthcare [11,12]. Lower SES is associated with reduced access to healthy food, safe spaces for physical activity, and preventive healthcare services, which collectively heighten diabetes risk and hinder disease management [13]. This uneven disease burden reflects inequities entrenched in behavioral, socioeconomic, and structural determinants of health. These persistent disparities highlight the need to better understand how diabetes affects emerging demographic groups whose health patterns remain largely unexamined [14,15].

Behavioral factors significantly influence the development and progression of diabetes, with various lifestyle-related predictors either increasing or reducing disease risk. Physical inactivity, obesity, tobacco use, poor dietary habits, and inadequate sleep have been consistently associated with increased diabetes incidence and complications [16,17] Conversely, protective behaviors such as regular physical activity, weight management, balanced nutrition, and moderate alcohol consumption have been shown to reduce the risk of diabetes and improve glycemic control for individuals at risk or living with the condition [18,19]. In addition to behavioral influences, genetic and heritable factors also contribute to diabetes susceptibility, and in multiracial populations, diverse ancestral backgrounds may create complex genotype–environment interactions that affect disease risk [20,21]. Multiracial adults may encounter overlapping disadvantage due to systemic social and institutional barriers related to economic instability, racial discrimination, and inconsistent healthcare experiences that constrain engagement in health-promoting behaviors and delay diagnosis. For example, ambiguous racial categorization and perceived exclusion in clinical encounters have been associated with lower screening rates and reduced trust in medical institutions [22]. Addressing both modifiable behaviors and systemic inequalities through targeted public health interventions remains essential to reducing the overall diabetes burden [13,23].

Recent demographic data show that multiracial Americans (identifying with two or more racial categories of White, Black or African American, Asian, Native Hawaiian, Other Pacific Islander, American Indian or Alaska Native, or some other race) represent one of the fastest-growing U.S. populations [24], increasing by 127% from 2010 to 2020 [25]. Despite extensive literature on diabetes among major racial and ethnic groups, multiracial adults remain underrepresented in epidemiological research. This population may experience unique social determinants of health, influenced by factors such as cultural diversity, discrimination, and variable access to health-promoting environments and healthcare [26,27]; yet, these factors remain understudied in the context of diabetes prevention and management. Understanding these distinctions is essential for identifying unique behavioral and sociodemographic predictors of diabetes specific to non-Hispanic multiracial adults.

The Behavioral Risk Factor Surveillance System (BRFSS) [28] offers a nationally representative dataset to explore these gaps, providing detailed information on health behaviors, access to care, and chronic disease prevalence [29]. This study examines behavioral and sociodemographic predictors of diabetes among non-Hispanic multiracial adults. The analysis focuses on whether established determinants such as body mass index (BMI), physical activity, smoking, alcohol consumption, self-rated health, insurance status, sex, age, and urbanicity demonstrate similar or distinct patterns compared with other racial/ethnic groups. It hypothesizes that diabetes correlating to multiracial groups will follow similar patterns. By integrating behavioral, demographic, and structural perspectives, this research seeks to generate empirical evidence to inform equitable, culturally responsive diabetes prevention and management strategies for an increasingly multiracial U.S. population.

## 2. Materials and Methods

### 2.1. Data Source and Study Design

This study employed a cross-sectional telephone survey to assess health-related risk behaviors, sociodemographic characteristics, chronic health conditions, and the use of preventive services among U.S. adults [29]. The BRFSS, administered by the U.S. Centers for Disease Control and Prevention (CDC) in collaboration with health departments from all 50 states, the District of Columbia, and U.S. territories, follows a standardized protocol to ensure methodological consistency across jurisdictions [30]. Since its inception in 1984, the BRFSS has expanded from 15 states to nationwide coverage, establishing itself as one of the most comprehensive and continuous public health surveillance systems [28].

In 2023, data collection included 48 states, the District of Columbia, Guam, Puerto Rico, and the U.S. Virgin Islands. Kentucky and Pennsylvania were excluded due to incomplete data collection. The final public dataset comprised 433,323 fully completed records, making it a robust source for population-level estimates of chronic disease prevalence and associated risk factors in the U.S. The BRFSS sampling methodology integrates both landline and cellular telephone interviews, targeting the non-institutionalized civilian population aged 18 years or older [28].

### 2.2. Sample Description

Each BRFSS record corresponds to one randomly selected telephone number from the survey’s sampling frame. All participating states met BRFSS standards for probability-based sampling of households with telephones. In 2023, 48 jurisdictions used a disproportionate stratified sample (DSS) for landline sampling, whereas Guam, Puerto Rico, and the U.S. Virgin Islands employed simple random sampling. The cellular telephone sample targeted adults aged 18 years and older residing in private homes or college housing who owned a working cellphone.

Participants were classified as having diabetes if they answered “yes” to the question: “Has a doctor, nurse, or other health professional ever told you that you had diabetes?” For this analysis, type 1 and type 2 diabetes were combined due to the unavailability of subtype-specific data. Individuals with gestational diabetes or borderline/pre-diabetes were excluded, as the research focused on identifying predictors of confirmed, ongoing diabetes cases among adults. Including those with gestational diabetes or borderline/pre-diabetes could lead to misclassification and bias. Weighted prevalence estimates were calculated using BRFSS raked sampling weights to generate state-level estimates that are representative of the U.S. adult population.

### 2.3. Study Variables

The variable names used are those reported in the BRFSS codebook and are shown in parenthesis. The primary outcome variable in this study was diabetes status (DIABETE4), coded as “yes” (1) for respondents with a self-reported physician-confirmed diagnosis of diabetes, and “no” (3) for those without. Individuals who reported gestational diabetes (2), pre-diabetes (4), or provided missing or invalid responses were excluded from the analysis through listwise deletion.

The independent variables included both behavioral and sociodemographic factors previously identified as relevant to diabetes risk. Behavioral factors included BMI (_BMI5CAT), categorized as underweight, normal weight, overweight, or obese; physical activity (_TOTINDA), defined as any leisure-time physical activity in the past 30 days; smoking status (_SMOKER3), indicating a lifetime history of smoking at least 100 cigarettes; depressive disorder (ADDEPEV3), based on history of depression diagnosis, self-reported general health (_RFHLTH) and alcohol consumption (DRNKANY6), measured as any alcohol intake in the past 30 days.

Sociodemographic variables included age category (_AGE65YR), sex (SEXVAR), race/ethnicity (_RACE), residence urbanicity (_URBSTAT), educational attainment (_EDUCAG), household income (_INCOMG1), and health insurance status (_HLTHPLN1). These variables were selected to investigate disparities in diabetes and to examine the contributions of behavioral and socioeconomic determinants to population-level risk [28].

### 2.4. Data Processing and Analysis

Descriptive statistics were used to summarize the characteristics of the study population. Categorical variables were presented as weighted frequencies and percentages. To ensure unbiased national estimates, all analyses accounted for the BRFSS’s complex survey design by applying sampling weights, strata, and primary sampling units. Bivariate associations between diabetes status and independent variables were evaluated using weighted logistic regression. Variables with *p*-values ≤ 0.20 in the bivariable analysis were included in the multivariable logistic regression to estimate adjusted odds ratios (AORs), 95% confidence intervals (CIs), and *p*-values. This liberal threshold was chosen to retain potential confounders that might gain significance after adjustment, consistent with recent epidemiologic modeling recommendations [31,32]. Statistical significance was set at *p* < 0.05. All analyses were performed in RStudio/https://www.r-project.org/ [33].

## 3. Results

### 3.1. Sociodemographic Characteristics

From the initial 2023 BRFSS sample of 10,125 non-Hispanic multiracial adults in the United States, 6429 participants met the inclusion criteria after excluding those with missing, incomplete, or non-applicable sociodemographic data. Adult participants aged 18–64 had a near equal distribution of males and females. Most of the participants had at least some college or a college degree and the most common household income range was $50,000 to <$100,000 per year. Nearly all respondents (94.73%) reported having current health insurance coverage, and the majority lived in urban areas (88.47%). See Table 1 for the full sociodemographic breakdown.

### 3.2. Health-Related Behaviors

The non-Hispanic multiracial adults had a near-even distribution of normal weight, overweight, and obese individuals. Most participants (78.32%) reported engaging in physical activity in the past 30 days, and 58.29% had never smoked. Over half of the respondents reported alcohol consumption in the past 30 days. Most participants rated their general health as good or better (78.60%), while 21.40% reported fair or poor health. Additionally, about a quarter of the participants reported a history of depressive disorder (Table 2).

**Figure 1:**
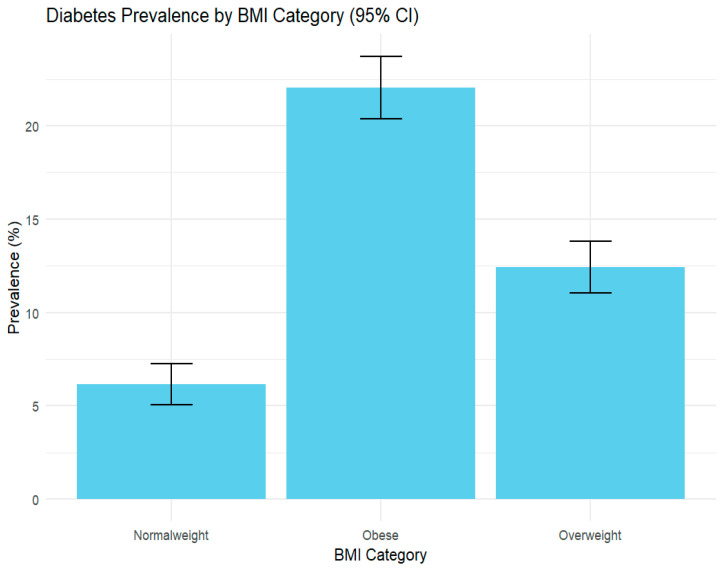
Weighted Prevalence of Diabetes by Body Mass Index (BMI) Category among Non-Hispanic Multiracial Adults in the United States: Behavioral Risk Factor Surveillance System, 2023 (*n* = 6429).

This study evaluated the weighted prevalence of diabetes across BMI categories among non-Hispanic multiracial adults. The analysis revealed a clear positive association between BMI and diabetes, with prevalence increasing from 6.2% (95% CI: 5.1–7.2) among adults with normal weight to 12.4% (95% CI: 11.0–13.8) among those overweight, and 22.1% (95% CI: 20.4–23.7) among obese adults. These findings underscore obesity as a key modifiable risk factor and demonstrate statistically significant differences in diabetes prevalence across BMI groups (Figure 1).

### 3.3. Predictors of Diabetes

Variables demonstrating statistically significant associations in bivariate analyses were subsequently retained for multivariable logistic regression. In the multivariable logistic regression analysis of non-Hispanic multiracial adults using datasets, several behavioral and sociodemographic factors were significantly associated with diabetes. Obesity (AOR = 4.16; 95% CI: 3.33, 5.23) and overweight status (AOR = 2.05; 95% CI: 1.62, 2.60) were strongly associated with higher odds of diabetes compared to individuals with normal weight. Additionally, fair or poor general health (AOR = 2.82; 95% CI: 2.38, 3.35) and older age (≥65 years; AOR = 3.08; 95% CI: 2.60, 3.65) were also associated with higher odds of diabetes. Having health insurance (AOR = 2.14; 95% CI: 1.35, 3.61) was strongly associated with higher odds of diabetes, likely indicating better access to healthcare and diagnosis. Male sex (AOR = 1.34; 95% CI: 1.15, 1.58) was associated with higher odds of diabetes, while engaging in physical activity (AOR = 0.76; 95% CI: 0.64, 0.90) and alcohol consumption (AOR = 0.55; 95% CI: 0.47, 0.65) were linked to lower odds of the disease. Smoking status did not show a statistically significant association (Table 3).

**Figure 2:**
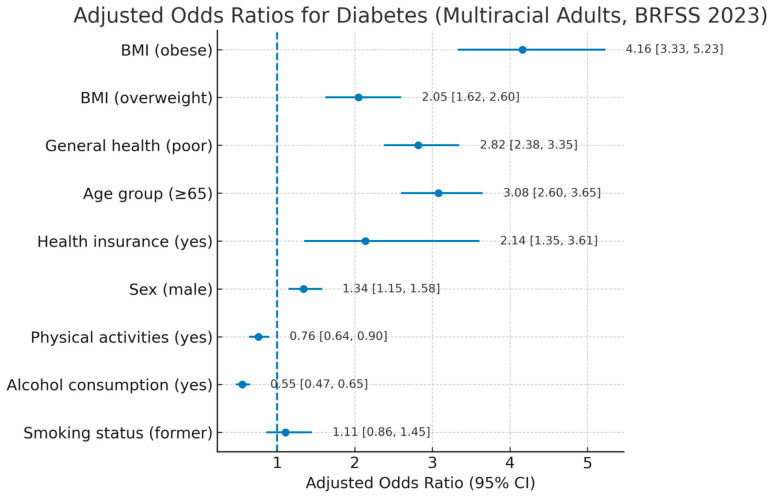
Forest plot showing adjusted odds ratios (AORs) and 95% confidence intervals (CIs) for behavioral and sociodemographic predictors of diabetes among non-Hispanic multiracial adults. Behavioral Risk Factor Surveillance System, 2023. Variables with AORs above 1 indicate increased odds, while those below 1 suggest lower odds.

The analysis shows that obesity, being overweight, poor general health, age (≥65 years), and having health insurance are significantly associated with higher odds of developing diabetes. In contrast, alcohol consumption and participation in physical activities are associated with lower odds of diabetes (Figure 2).

## 4. Discussion

This study aimed to fill a significant gap in U.S. diabetes research, specifically the scarcity of population-based evidence regarding behavioral and sociodemographic predictors of diabetes among non-Hispanic multiracial adults. Utilizing the nationally representative 2023 BRFSS data, we explored whether the established risk and protective factors identified in the broader U.S. population apply to this understudied group. Our findings indicate that BMI, age, sex, physical activity, smoking, alcohol use, general health status, and health insurance affect this population similarly to how they affect non-multiracial populations. This analysis is timely, considering the increasing national prevalence of diabetes and the well-documented geographic and demographic disparities in both risk and outcomes [6,16].

In this study, we found that both obesity and overweight status are associated with significantly higher odds of developing diabetes compared to individuals with normal weight. This finding aligns with recent research emphasizing the critical role of adiposity in the pathogenesis of type 2 diabetes (T2D) through mechanisms such as insulin resistance, ectopic fat deposition, and pro-inflammatory signaling [12,34]. Furthermore, recent reviews and meta-analyses confirm that the transition from normal weight to overweight and obesity greatly heightens the risk of T2D, and that worsening adiposity further increases this risk [35,36]. Our estimates are therefore consistent with these findings, underscoring the importance of weight management in preventing diabetes within multiracial populations.

In our study, participants who reported fair or poor general health had higher odds of diabetes than those reporting good or better health, aligning with recent evidence that links self-rated health to the burden of metabolic disease and its complications [37,38]. General health encompasses symptoms, comorbidities, and functional abilities, likely serving as both a correlation and a proxy for severity. This may help clarify its strong association in cross-sectional data. Additionally, the observed effect is consistent with national surveillance data, which shows that adults with diabetes experience greater cardiometabolic comorbidity and increased care needs [39,40].

In our study, we found that being physically active in the past 30 days was inversely associated with diabetes, aligning with established protective effects of exercise on glucose metabolism [41], insulin sensitivity, and weight management [42,43]. The direction and magnitude of our findings are consistent with recent reviews highlighting the benefits of both aerobic and resistance training [44]. This supports the promotion of accessible and culturally responsive activity programs for multiracial communities [45,46].

In this study, we found that alcohol consumption was inversely associated with diabetes. Contemporary prospective evidence increasingly supports a pattern-dependent relationship, in which light-to-moderate regular drinking is linked to lower diabetes risks, while heavier intake is harmful [47,48]. This negative association may be due to participants being more likely to reflect the light-to-moderate drinking population [49,50]; however, caution is warranted due to potential residual confounding factors, such as socioeconomic or dietary differences, and the possibility of reverse causation, where individuals may abstain from alcohol due to illness [51]. Due to the cross-sectional nature of this study, causation between alcohol consumption and diabetes cannot be inferred. These results should not be interpreted as a recommendation to begin alcohol use; instead, they should be viewed in the context of the existing literature [52].

Our findings suggest that having health insurance was associated with higher odds of diagnosed diabetes, likely due to increased detection rather than a direct causal relationship, increased disease incidence, or greater prevalence. Individuals with insurance are more likely to receive regular screenings, routine checkups, and timely diagnoses compared to those without coverage [16,53]. Recent federal reports also show that adults with diabetes tend to have higher rates of insurance coverage, underscoring its critical role in facilitating access to diagnostic and treatment services [54]. Furthermore, these findings highlight the diagnostic gap among uninsured populations and the urgent need for improved healthcare access to tackle issues of underdiagnosis and delayed treatment. Addressing these barriers through expanded healthcare coverage and culturally competent services is essential to reduce underdiagnosis and improve diabetes management among non-Hispanic multiracial adults [55,56].

In our study, we found that older age (≥65 years) was significantly associated with an increased risk of diabetes, aligning with national trends that indicate cumulative metabolic dysregulation with aging [57,58]. Implementing interventions focused on early screening and lifestyle changes during early or midlife could help mitigate the progression to diabetes in older adulthood [59].

We observed sex differences, with males having higher odds of diabetes than females after adjustments were made. Recent global and U.S. analyses have reported that men are diagnosed at younger ages and at lower BMI thresholds compared to women [60,61]. This suggests a sex-specific susceptibility and potentially different pathways, such as visceral adiposity and hormonal factors [62,63]. Our findings extend these observations to multiracial adults, emphasizing the importance of sex-tailored screening thresholds and counseling.

In our study, smoking status did not show a significant association with diabetes after adjusting for other variables. This finding contrasts with previous studies that have linked smoking to impaired glucose metabolism and insulin resistance [64,65]. The lack of statistical significance here may be due to the relatively small size of the smoking subgroups within this multiracial sample or residual confounding [66].

### Strengths and Limitations of Study

This study has several strengths, including a large, nationally representative dataset and standardized data collection procedures, which enhance the generalizability and reliability of the findings for non-Hispanic multiracial adults in the U.S. The inclusion of a wide range of sociodemographic, behavioral, and health-related variables allowed for a thorough assessment of diabetes predictors within this population. Additionally, the use of survey weights provided accurate estimates that accounted for the complex sampling design. Furthermore, the multivariable logistic regression model demonstrated strong analytical robustness, effectively adjusting for potential confounders and ensuring reliable estimation of independent associations.

However, there are notable limitations. The cross-sectional design restricts the ability to draw causal inferences, and reliance on self-reported measures for diabetes diagnosis and behavioral factors may introduce bias, including recall or social desirability bias. Of particular note, self-reported weight and height can be unreliable, leading to inaccurate BMI calculations. Furthermore, the inability to differentiate between type 1 and type 2 diabetes limits the specificity of the findings. Future research should explore these multilevel influences using longitudinal data and incorporate genetic, dietary, and neighborhood-level variables to better capture heterogeneity within the multiracial population. Studies should also distinguish between diabetes types to account for their differing etiologies, risk factors, and demographic characteristics. Additionally, interactions between the study variables are not accounted for in this model and may limit the study results. The BRFSS dataset does not include detailed dietary intake information such as refined sugar or saturated fat consumption; therefore, diet-related influences on diabetes risk could not be directly assessed. However, existing literature consistently supports the role of diet quality in diabetes prevention and management. Lastly, residual confounding from unmeasured variables such as diet and family history may still exist, even after multivariable adjustments.

## 5. Conclusions

Obesity, overweight status, older age, male sex, poor or fair general health, and having health insurance were associated with increased odds of diabetes. In contrast, physical activity and alcohol consumption in the past 30 days were linked to lower odds. These findings confirm similar diabetes risk and protective factors among non-Hispanic multiracial adults despite the disproportionate burden and highlight the need for targeted, culturally responsive interventions. Such interventions should prioritize weight management, promote physical activity, facilitate early screening, and ensure equitable access to preventive care. From a public health standpoint, the results reinforce the importance of implementing community-based education programs, culturally tailored lifestyle interventions, and policy initiatives that address the broader social determinants of health such as food security and safe environments for physical activity to mitigate diabetes risk among multiracial adults. By providing subgroup-specific evidence, this study offers timely, actionable insights to guide public health practitioners, policymakers, and researchers in designing inclusive diabetes prevention and management strategies that advance health equity in an increasingly diverse U.S. population.

## Figures and Tables

**Table 1:** Sociodemographic Characteristics of non-Hispanic Multiracial Adults in the United States: Behavioral Risk Factor Surveillance System, 2023 (*n* = 6429).

Characteristic	Frequency (n)	Percentage (%)
Age group		
18–64 years	4992	77.65
≥65 years	1437	22.35
Sex of participants		
Male	3279	51.00
Female	3150	49.00
Educational attainment		
Did not graduate high school	284	4.42
High school diploma	1515	23.57
Some college/technical school	2026	31.51
College graduate	2604	40.50
Annual household income		
<$15,000	393	6.11
$15,000 to <$25,000	610	9.49
$25,000 to <$35,000	715	11.12
$35,000 to <$50,000	873	13.58
$50,000 to <$100,000	1987	30.91
$100,000to <$200,000	1367	21.26
≥$200,000	484	7.53
Health insurance status		
No	339	5.27
Yes	6090	94.73
Residence Urbanicity		
Urban	5688	88.47
Rural	741	11.53

**Table 2:** Health-Related Behaviors among non-Hispanic Multiracial Adults in the United States: Behavioral Risk Factor Surveillance System, 2023 (*n* = 6429).

Characteristic	Frequency (n)	Percentage (%)
Body mass index (BMI)		
Normal weight	1901	29.57
Overweight	2188	34.03
Obese	2340	36.40
Physical activity in past 30 days		
Yes	5035	78.32
No	1394	21.68
Smoking status		
Current smoker	753	11.71
Former smoker	1928	30.00
Never smoked	3748	58.29
Alcohol consumption (past 30 days)		
Yes	3460	53.82
No	2969	46.18
General health status		
Good or better	5053	78.60
Fair or poor	1376	21.40
History of depressive disorder		
Yes	1763	27.42
No	4666	72.58

**Table 3:** Multivariable Logistic Regression Results for Diabetes by Behavioral and Socioeconomic among non-Hispanic Multiracial Adults in the United States: Behavioral Risk Factor Surveillance 2023 (*n* = 6429).

Characteristic	AOR	95% CI	*p*-Value
Body mass index			
Normal weight			
Overweight	2.05	1.62, 2.60	<0.001
Obese	4.16	3.33, 5.23	<0.001
General health			
Good or better			
Fair or poor	2.82	2.38, 3.35	<0.001
Age categories			
Age 18 to 64			
Age 65 or older	3.08	2.60, 3.65	<0.001
Health insurance			
No			
Yes	2.14	1.35, 3.61	0.002
Physical activity in past 30 days			
No			
Yes	0.76	0.64, 0.90	0.002
Sex of respondent			
Female			
Male	1.34	1.15, 1.58	<0.001
Smoking status			
Current smoker			
Former smoker	1.11	0.86, 1.45	0.4
Never smoked	1.0	0.77, 1.29	>0.9
Alcohol consumption (past 30 days)			
No			
Yes	0.55	0.47, 0.65	<0.001

Abbreviations: CI = Confidence Interval, AOR = Adjusted Odds Ratio.

## Data Availability

Researchers seeking raw data to support their non-commercial projects can obtain it by requesting access from the corresponding author.
